# Inequalities in health outcomes of SARS-CoV-2 infection by migration status in Barcelona, Spain

**DOI:** 10.3389/fpubh.2023.1297025

**Published:** 2024-01-08

**Authors:** Valeria Pérez-Muto, Maria Jesús Bertran, Lourdes Barón-Miras, Isabel Torá-Rocamora, Juan José Gualda-Gea, Anna Vilella

**Affiliations:** ^1^Department of Preventive Medicine and Epidemiology, Clinical Institute of Medicine and Dermatology (ICMiD), Hospital Clínic, Barcelona, Spain; ^2^ISGlobal, Hospital Clínic, Universitat de Barcelona, Barcelona, Spain; ^3^Department of Medicine, Faculty of Medicine and Health Sciences, University of Barcelona, Barcelona, Spain

**Keywords:** SARS-CoV-2, population health, syndemic, emigrants and immigrants, health inequities, Spain

## Abstract

**Background:**

Migrants are a vulnerable population at risk of worse health outcomes due to legal status, language barriers, and socioeconomic and cultural factors. Considering the conflicting literature on the subject, it is important to further explore the extent and nature of these inequalities.

**Objective:**

The aim of this study is to compare health outcomes associated with SARS-CoV-2 infection between Spanish native and migrant population living in Barcelona.

**Methods:**

Observational retrospective cohort study including all adult cases of SARS-CoV-2 infection who visited a tertiary hospital in Barcelona between the 1st March 2020 and the 31st March 2022. We established the following five health outcomes: the presence of symptomatology, hospitalisation, intensive care unit admission, use of mechanical ventilation, and in-hospital 30-day mortality (IHM). Using Spanish natives as a reference, Odds Ratios (OR) with 95% confidence interval (95%CI) were calculated for migrants by multivariate logistic regression and adjusted by sociodemographic and clinical factors.

**Results:**

Of 11,589 patients (46.8% females), 3,914 were born outside of Spain, although 34.8% of them had legal citizenship. Most migrants were born in the Americas Region (20.3%), followed by other countries in Europe (17.2%). Migrants were younger than natives (median 43 [IQR 33–55] years vs. 65 [49–78] years) and had a higher socioeconomic privation index, less comorbidities, and fewer vaccine doses. Adjusted models showed migrants were more likely to report SARS-CoV-2 symptomatology with an adjusted OR of 1.36 (95%CI 1.20–1.54), and more likely to be hospitalised (OR 1.11 [IC95% 1.00–1.23], *p* < 0.05), but less likely to experience IHM (OR 0.67 [IC95% 0.47–0.93], *p* < 0.05).

**Conclusion:**

Characteristics of migrant and native population differ greatly, which could be translated into different needs and health priorities. Native population had higher odds of IHM, but migrants were more likely to present to care symptomatic and to be hospitalised. This could suggest disparities in healthcare access for migrant population. More research on health disparities beyond SARS-CoV-2 in migrant populations is necessary to identify gaps in healthcare access and health literacy.

## 1 Introduction

The SARS-CoV-2 pandemic has been one of the greatest public health challenges in the era of globalisation. Its impact goes beyond health and across borders, affecting the economy, employment, education, and social interactions. The pandemic has also made more evident than ever before important inequalities through disproportionate health effects on some populations. The older adult, high-risk workers, people living in poverty, and displaced communities are among those most affected (1, 2).

Health inequalities have been harder to tackle during the pandemic due to its continuous detrimental effect on the social determinants of already vulnerable groups (3). This has given way to a syndemic, with the SARS-CoV-2 crisis converging with other adverse conditions to produce worse health outcomes in certain population groups, such as migrant communities (4). Migration and the displacement of people is one of the global priority challenges. The United Nations (UN) reported more than 244 millions of international migrants, including more than 20 million refugees (4).

During the different stages of the migratory process, people face precarious circumstances, such as food insecurity, poverty, violence, and issues with legalisation of their migratory status, that can gravely affect their physical and mental health (4). Migration is an important social determinant by itself, but it also interacts with other factor such as gender, labour, accommodation conditions, socioeconomic status, or ethnicity, to exacerbate health vulnerability, whether it is biological or due to health literacy, access to healthcare, or conflictive relations with state institutions. This could translate into worse health outcomes even though migrant population tends to be younger and healthier than autochthonous population (4, 5).

Despite being a developed country with universal access to quality healthcare, Spain was one of the first and most affected European countries to face the SARS-CoV-2 pandemic. According to the last situation update by the Spanish Ministry of Health, until the end of June, 2023, there have been almost 14 million reported cases of SARS-CoV-2 infection in Spain, as well as more than 121,000 deaths (6). Catalonia has been one of the Autonomous Communities most affected by the pandemic while having a significant proportion of migrant population. In Barcelona, Catalonia’s largest and most cosmopolitan city, it is estimated that more than 350.000 people (20% of its population) were born outside of Spain, mainly in Latin America, Morocco, China, Pakistan, and other European countries (7).

European studies have described heterogeneous results; while some found no significant differences in health outcomes due to SARS-CoV-2 infection between native and immigrant population, others reported higher risk of infection and hospitalisation rates (8–19). There have been a few studies from Spain, but most were performed at the beginning of the pandemic and had also conflicting results; some concluded there were no significant differences in ICU admission or mortality, while others stated that some groups of immigrants had higher risk of ICU admission (20–24).

This inconsistency in literature, combined with a systematic neglect in registering variables such as country of birth or ethnicity, hinders adequate analysis of the impact migration status has on various health outcomes. Analysis of this data can enlighten us to better target policies and interventions to reinforce assistance during health crisis in an equitable way. The main objective of this study was to compare health outcomes associated with SARS-CoV-2 infection between Spanish native and migrant population living in Barcelona.

## 2 Materials and methods

### 2.1 Study design and setting

This was an observational retrospective cohort study of all SARS-CoV-2 infections in adult patients who were visited at Hospital Clinic Barcelona, a large university tertiary hospital, between the 1st of March of 2020 and the 31st of March of 2022. The patients were seen at the Emergency Department, in outpatient clinics, during hospitalisation (hospital-onset infections), or they could have been diagnosed with SARS-CoV-2 incidentally, as part of pre-operative tests. Therefore, not all patients were symptomatic, and a SARS-CoV-2 infection was not necessarily the reason why patients were visited.

Infection by SARS-CoV-2 was confirmed either by Polymerase Chain Reaction or by Rapid Antigen Test, even if it was a self-administered test with a positive result reported by the patient. We excluded patients under 18 years of age, hospital workers, and patients whose relevant information could not be retrieved. Repeated positive SARS-CoV-2 tests in the same patient were only considered as reinfection if 90 days or more had passed since the first positive test. Since patients could have had two or more SARS-CoV-2 infections during the study’s period, each infection episode is considered an individual case. Henceforth, we use the terms “patients” and “cases” indifferently, although we are referring to a SARS-CoV-2 infection episode.

### 2.2 Data collection

Data was obtained from the routine surveillance system database of the Preventive Medicine and Epidemiology Department. This database received real-time information about positive SARS-CoV-2 test results from the Department of Microbiology and manual registration of SARS-CoV-2 infection by healthcare teams in case the patient reported a positive ART or an external PCR result. Complementary information about cases was completed by the surveillance team using data from hospital records. We included information about sociodemographic characteristics (age, sex, Basic Health Area (BHA) where the patient lives, country of birth, and nationality), relevant medical history and comorbidities, SARS-CoV-2 vaccination, symptomatology at the time of the visit, and health outcomes such as duration of hospitalisation, Intensive Care Unit (ICU) admission, mechanical ventilation and mortality.

### 2.3 Variable definitions

We considered five unfavourable health outcomes associated with SARS-CoV-2 infection among patients of the cohort. These included the presence of symptomatology at the time of hospital visit, hospitalisation, in-hospital 30-day mortality, ICU admission, and use of mechanical ventilation (MV) during SARS-CoV-2 infection. ICU admission and MV information could only be obtained for patients hospitalised at our centre. In-hospital 30-day all-cause mortality was considered for patients with SARS-CoV-2 who had been hospitalised 30 days or less at the time of in-hospital death or if they had been diagnosed with a SARS-CoV-2 infection in the 30 days before their death, if it was a hospital-onset infection. Although a distinction is made between orotracheal intubation (OTI) and non-invasive mechanical ventilation (NIMV), for the multivariate logistic regression of MV use, we have dichotomised the variable and considered both OTI and NIMV as MV use.

Our exposure of interest was migration status as a dichotomous variable (migrant vs. native Spanish). We have defined migrant status as being born outside of Spain (being a non-native of Spain) irrespective of legal nationality. Native Spanish patients were considered as a reference category. We also analysed health outcomes disaggregating by region of birth. We decided to use the World Health Organization classification of the world’s regions due to its widespread use and recognition: African Region, Region of the Americas, South-East Asian Region, European Region, Eastern Mediterranean Region, and Western Pacific Region.

We considered the following covariates: socio-demographic and geographical factors, SARS-CoV-2 vaccination, epidemiological period of contagion, presence of symptoms, a constructed comorbidity score and seven individual comorbidities. Age was described as a continuous variable, but we also categorised it into quartiles (18–40, 41–57, 58–73, and >73 years old). The Catalonian socioeconomic deprivation index (SDI) based on the BHA of residence used in this study was created by the Healthcare Quality and Evaluation Agency of Catalonia (AQuAS) (25). There was an important proportion of missing data which we could not entirely attribute to randomness, which is why we decided not to adjust the models for these variables.

We selected seven comorbidity groups that may be associated with the risk of SARS-CoV-2 infection and with higher mortality due to SARS-CoV-2: cardiovascular diseases (CVD), cardiovascular risk factors (CVRF) (obesity, dyslipidaemia, hypertension, alcohol consumption and/or smoking), diabetes mellitus (DM), chronic respiratory diseases (CRD), immunosuppression (solid organ transplant, hematopoietic transplant, cancer, and other types of immunosuppression), chronic kidney disease (CKD), and hepatic disease. As information on comorbidities was not sufficiently specific to use a known comorbidity index, we constructed our own comorbidity score by adding the number of each of these groups of comorbidities (26, 27). The categories of this score are 0, 1, 2 and ≥ 3 comorbidities. For the vaccination variable, we considered the person fully vaccinated if two or more doses were registered and the last dose was administered at least 14 days before the SARS-CoV-2 infection diagnosis.

We considered six pandemic epidemiological periods consistent with infection dynamics described in Spain: first period from 1st March 2020 to 31st May 2020; second period from 1st June 2020 to 30th November 2020; third period from 1st December 2020 to 28th February 2021; fourth period from 1st March 2021 to 30th June 2021; fifth period from 1st July 2021 to 15th October 2021; sixth period from 16th October 2021 to 31st March 2022.

### 2.4 Statistical analysis

We carried out a descriptive analysis of all relevant sociodemographic variables for the entire population and for the native and migrant groups. Continuous variables were summarised as means with standard deviation or medians with interquartile ranges (IQR) if not normally distributed Categorical variables were described as absolute frequency (n) and relative frequency (in percentages). For the comparative analysis between the native and the migrant group, we used either Chi^2^ test or Fisher test for qualitative variables, and t-student or Mann–Whitney U test for quantitative variables.

Bivariate and multivariate analysis was performed with a subset of complete cases in the variables that we would theoretically fit into the model. We used logistic regression models only adjusted by sex and age quartile to obtain minimally adjusted Odds Ratios (mOR) and their 95% confidence intervals (95% CI) for each of the five outcomes. Multivariate logistic regression models were also performed to obtain a fully adjusted Odds Ratios (aOR) with 95% CI. Relevant covariates additionally to sex and age quartiles were first selected based on theoretical hypotheses; then the model was further refined using backward stepwise selection, considering the significance of each variable and the performance of Likelihood Ratio Tests (LRT) and Akaike’s criterion (AICc) values. The R-project® statistical program in its 4.1 version was used for data processing and analysis. A value of *p* < 0.05 was statistically significant (28). Microsoft Excel® was also used for the odds ratio Forest plots.

## 3 Results

### 3.1 Descriptive analysis of the population

**Figure 1:**
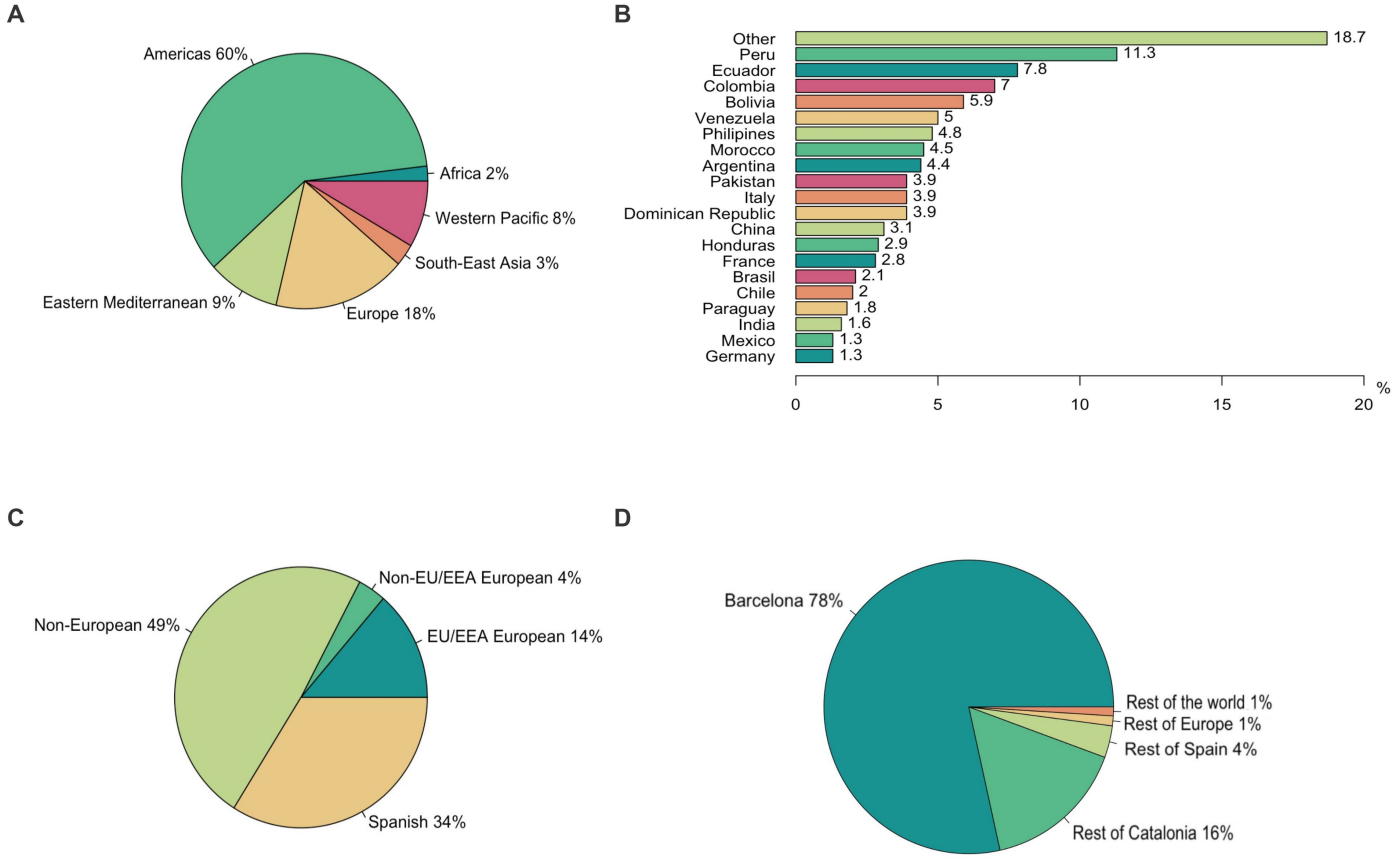
Geodemographic characteristics of non-native patients. **(A)** Distribution of migrant patients by country of birth (only the twenty most frequent countries are displayed). **(B)** Distribution of migrants by WHO region of birth. **(C)** Distribution of migrants by nationality type (distinction made between Spanish, EU/EEA Europeans, non-EU/EEA Europeans, and non-Europeans). **(D)** Distribution of migrants by place of residence at the time of the COVID-19 infection.

We included 11,589 SARS-CoV-2 infection cases between the 1st of March 2020 and the 31st of March 2022. A total of 3,914 (33.8%) SARS-CoV-2 infections were diagnosed in migrants, but 34% (Figure 1C) of them had Spanish nationality and 49.4% had a long-term or short-term visa, with the remaining 613 migrant patients being either tourists or undocumented migrants. Additionally, another 531 migrants (14%) had a European Union/European Economic Area (EU/EEA) nationality (Figure 1C). Most migrant patients were born in the Americas Region (60%), followed by the European Region (18%) (Figure 1B). A more detailed distribution of migrants by country of birth, WHO Region of birth, type of nationality, and place of residence can be found in Figures 1A–D.

The median of age was 57 years [IQR 40–73] and 46.8% were female. The descriptive analysis of all variables in native and migrant populations can be found in Table 1. A higher percentage of migrants compared to Spanish were female (49.6% vs. 44.9%). Migrants were significantly younger than Spanish with a median of 43 years [IQR 33–55] vs. 65 years [IQR 49–78]. Nearly 80% of migrants were younger than 58 years of age, compared to 36.7% of Spanish in those age categories.

**Table 1:** Sociodemographic, clinical, and epidemiologic characteristic of the total population, comparing Spanish and migrant groups.

Variable	Total^1^	Spanish natives^1^	Migrants^1^	*p*-value^2^
Total	11,589	7,675 (66.2%)	3,914 (33.8%)	
Female	549 (46.8)	3,446 (44.9)	1,973 (49.6)	<0.001
Age (years)	57 [40–73]	65 [49–78]	43 [33–55]	<0.001
Age categories				
18–40 years	2,922 (25.2)	1,211 (15.8)	1,711 (43.7)	<0.001
41–57 years	2,989 (25.8)	1,603 (20.9)	1,386 (35.4)	
58–73 years	2,919 (25.2)	2,279 (29.7)	640 (16.34)	
>73 years	2,759 (23.8)	2,582 (33.6)	177 (4.5)	
Registered with Catalonian GP	10,943 (94.4)	7,444 (97.0)	3,499 (89.4)	<0.001
Socioeconomic privation index	23.0 [10.7–35.4]	19.3 [10.7–35.4]	25.6 [13.3–40.1]	<0.001
Socioeconomic privation index by categories				
0–12.0%	2,422 (20.9)	1,766 (23.0)	656 (16.8)	<0.001
12.1–25.0%	2,669 (23.0)	1,831 (23.9)	838 (21.4)	
25.1–40.0%	2,359 (20.4)	1,508 (19.6)	851 (21.7)	
40.1–100%	2,006 (17.3)	1,202 (15.7)	804 (20.5)	
*Missing*	2,133 (18.4)	1,368 (17.8)	765 (19.5)	
Comorbidities				
CV risk factors	4,832 (41.7)	3,773 (49.2)	1,059 (27.1)	<0.001
Cardiovascular disease	1,822 (15.7)	1,614 (21.0)	208 (5.3)	<0.001
Diabetes mellitus	1,525 (13.2)	1,207 (15.7)	318 (8.1)	<0.001
Respiratory disease	1,507 (13.0)	1,216 (15.8)	291 (7.4)	<0.001
Immunosuppression	1,927 (16.6)	1,558 (20.3)	369 (9.4)	<0.001
Renal disease	1,012 (8.7)	871 (11.3)	141 (3.6)	<0.001
Hepatic disease	484 (4.2)	364 (4.7)	120 (3.1)	<0.001
*Missing*	748 (6.5)	529 (6.9)	219 (5.6)	
Comorbidity score				
0	4,288 (37.0)	2,163 (28.2)	2,125 (54.3)	<0.001
1	2,825 (24.4)	1,875 (24.4)	950 (24.3)	
2	1,884 (16.2)	1,486 (19.4)	398 (10.2)	
≥3	1,844 (15.9)	1,622 (21.1)	222 (5.7)	
*Missing*	748 (6.5)	529 (6.9)	219 (5.6)	
SARS-CoV-2 vaccination				
<2 doses	8,981 (77.5)	5,669 (73.9)	3,312 (84.6)	<0.001
≥2 doses	2,608 (22.5)	2,006 (26.1)	602 (15.4)	
SARS-CoV-2 epidemiological period^3^				
1st period	2,123 (18.3)	1,576 (20.5)	547 (14.0)	<0.001
2nd period	1,621 (14.0)	881 (11.5)	740 (18.9)	
3rd period	1,891 (16.3)	1,434 (18.4)	457 (11.7)	
4th period	1,280 (11.0)	812 (10.6)	468 (12.0)	
5th period	1,623 (14.0)	845 (11.0)	778 (19.9)	
6th period	3,051 (26.3)	2,127 (27.7)	924 (23.6)	
Symptoms	9,544 (82.4)	6,186 (80.6)	3,358 (85.8)	<0.001
Days from symptoms onset to hospital visit	4 [2–8]	4 [2–8]	4 [2–7]	0.654
Pneumonia at the time of hospital visit				
Yes	4,060 (35.0)	2,764 (36.0)	1,296 (33.1)	<0.001
*Missing*	1,111 (9.6)	816 (10.6)	295 (7.5)	
Hospitalisation				
Hospital Clínic de Barcelona (HCB)	5,942 (51.3)	4,314 (56.2)	1,628 (41.6)	<0.001
Other hospital	213 (1.8)	163 (2.1)	50 (1.3)	
No hospitalisation	5,434 (46.9)	3,198 (41.7)	2,236 (57.1)	
Duration of hospital stay (days) (n = 5942)	9 [6–16]	10 [6–17]	8 [5–12]	<0.001
ICU admission in HCB patients (n = 5942)	1,463 (24.6)	1,070 (24.8)	393 (24.1)	0.620
Duration of ICU stay (days) (n = 1463)	6 [3–12]	6 [3–12]	6 [3–11]	0.324
Mechanical ventilation in HCB hospitalised patients (*n* = 5942)				
Orotracheal intubation	465 (7.8)	342 (7.9)	123 (7.6)	0.863
Non-invasive ventilation	367 (6.2)	264 (6.1)	103 (6.3)	
No ventilation	5,110 (86.0)	3,708 (86.0)	1,402 (86.1)	
In-hospital 30-day mortality				
(% of all patients)	640 (5.5)	585 (7.6)	55 (1.4)	<0.001
(% of HCB hospitalised patients)	640 (10.8)	585 (13.6)	55 (3.4)	<0.001
Days from admission to death	8 [4–15]	9 [4–15]	7 [2.5–16]	0.486
Immediate death at hospital arrival^4^	50 (7.8)	43 (7.4)	7 (12.7)	0.183

1 *n* (%); Median [IQR].

2 Pearson’s Chi-squared test; Wilcoxon-Mann–Whitney test.

3 SARS-CoV-2 epidemiologic periods: from 25th February 2020 to 31st May 2020; from 1st June 2020 to 30th November 2020; from 1st December 2020 to 28th February 2021; from 1st March 2021 to 30th June 2021; from 1st July 2021 to 15th October 2021; and from 16th October 2021 to 31st March 2022.

4 Fisher test.

The Catalonian SDI associated to the BHA of residence was significantly higher (more deprivation) for migrants (median of 25.6 [IQR 13.3–40.1] vs. 19.25 [IQR 10.7–35.4]), although there was an important proportion of missing information (18.4%). At the time of the hospital visit, 97.0% of native patients had been registered with a general practitioner (GP) in Catalunya and had a sanitary card that allowed them access to healthcare services without charge; however, only 89.4% of migrant population had been registered.

There were significant differences in the prevalence of each individual comorbidity between both groups (Table 1); the proportion of patients with each of the comorbidities was always larger for Spanish. The most prevalent type of comorbidity in both groups was cardiovascular risk factors (49.2% in natives and 27.1% in migrants). The number of comorbidities also varied widely, showing statistically significant differences (*p* < 0.001), with 54.3% of migrant patients vs. only 28.2% natives having no comorbidities. A considerable percentage of Spanish native patients had three or more comorbidities (21.1%), while only 5.7% of migrant patients were in this category.

**Figure 2:**
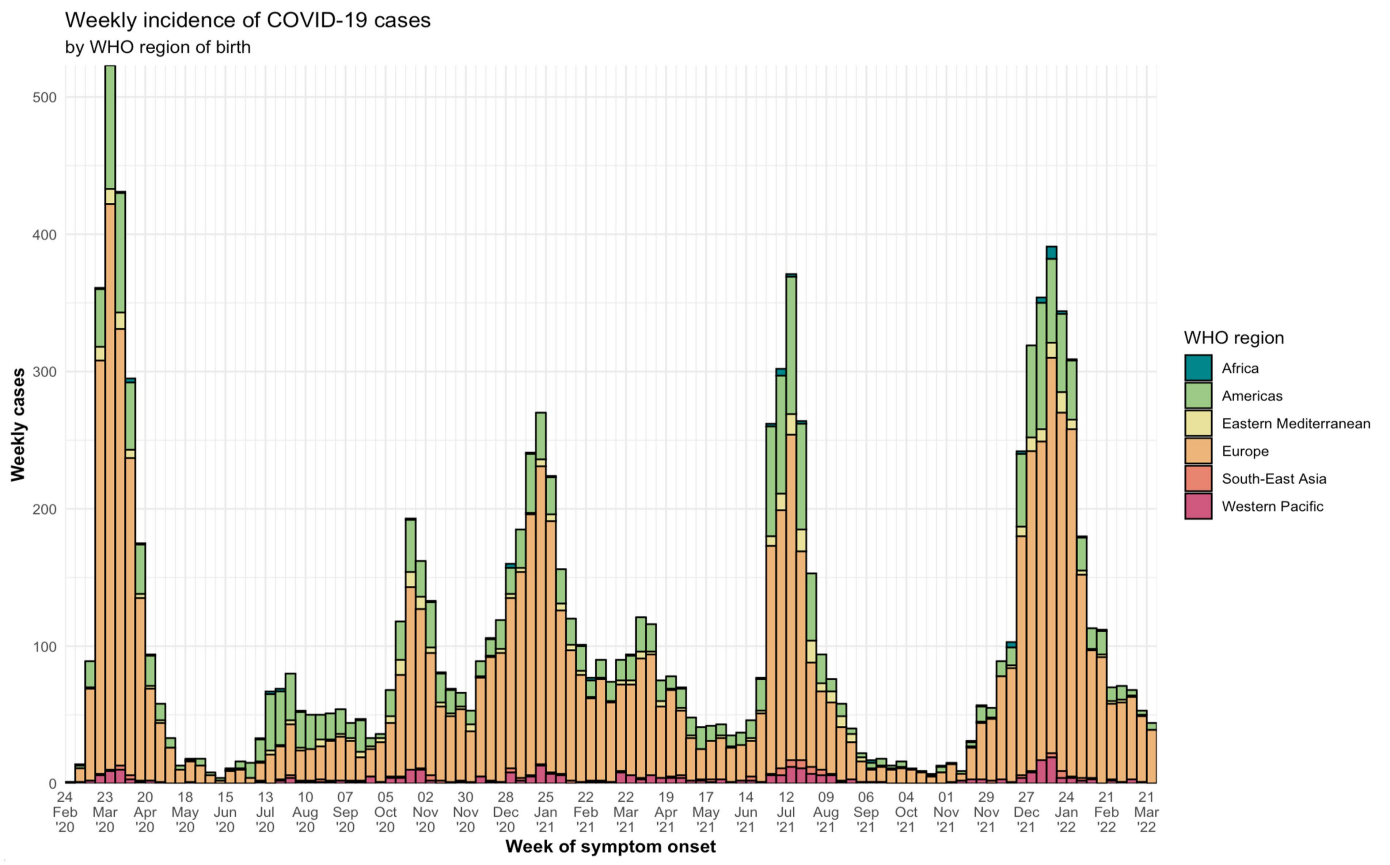
Weekly cases of COVID-19 cases by WHO Region of birth from the 1st of March, 2020 until the 31st of March, 2022. It is possible to identify the six periods of the pandemic according to epidemiological waves in Spain: from 25th February 2020 to 31st May 2020; from 1st June 2020 to 30th November 2020; from 1st December 2020 to 28th February 2021; from 1st March 2021 to 30th June 2021; from 1st July 2021 to 15th October 2021; and from 16th October 2021 to 31st March 2022.

Full vaccination at the time of infection was only present in 26.1% of Spanish patients and 15.4% of migrants (*p* < 0.001). There were also differences in the distribution of both groups through the six epidemiological periods. Although the number of native cases were always higher than the number of migrant cases throughout all periods, during the 2nd, 5th, and 6th periods there was a high concentration of migrant patients compared to other periods (Table 1). A weekly incidence graphic by region of birth can be found in Figure 2.

There was a higher percentage of migrant patients presenting symptoms at the time of the hospital’s visit compared to Spanish patients (85.8% vs. 80.6%). However, there were no statistical differences in the duration of symptoms until diagnosis (median of 4 [IQR 2–8] days for Spanish and of 4 [IQR 2–7] days for migrants, *p* = 0.654). More native patients were clinically or radiologically diagnosed with pneumonia during the initial healthcare contact (36.0% vs. 33.1%, *p* < 0.001). However, it is important to consider the large proportion of missing information for this variable (9.6%).

A total of 6,155 (53.1%) cases were hospitalised during a SARS-CoV-2 infection, although 213 ended up being admitted to another centre and were lost at follow-up. Out of the remaining 5,942 cases hospitalised at our centre, 13.7% had no symptoms compatible with SARS-CoV-2 infection at the time of admission. We admitted a lower proportion of migrant patients compared with native patients (41.6% vs. 56.2%) and the length of stay was also shorter for migrants (8 days [IQR 5–12] vs. 10 days [IQR 6–17], *p* < 0.001).

In-hospital 30-day all-cause mortality was registered in 3.4 and 13.6% of migrant and native hospitalised patients, respectively (*p* < 0.001); however, there were no statistical differences in the time between admission (or diagnosis if it was an in-hospital onset infection) and death for both groups (7 days [IQR 2.5–16] vs. 9 days [4–15], *p* = 0.486). We also compared immediate deaths, defined as death of a patient in the 24 h of hospital arrival (death at the Emergency Department), and no statistical difference were found (12.7% in migrants vs. 7.4% in natives, *p* = 0.183).

### 3.2 Presence of symptoms at the time of hospital visit

**Figure 3:**
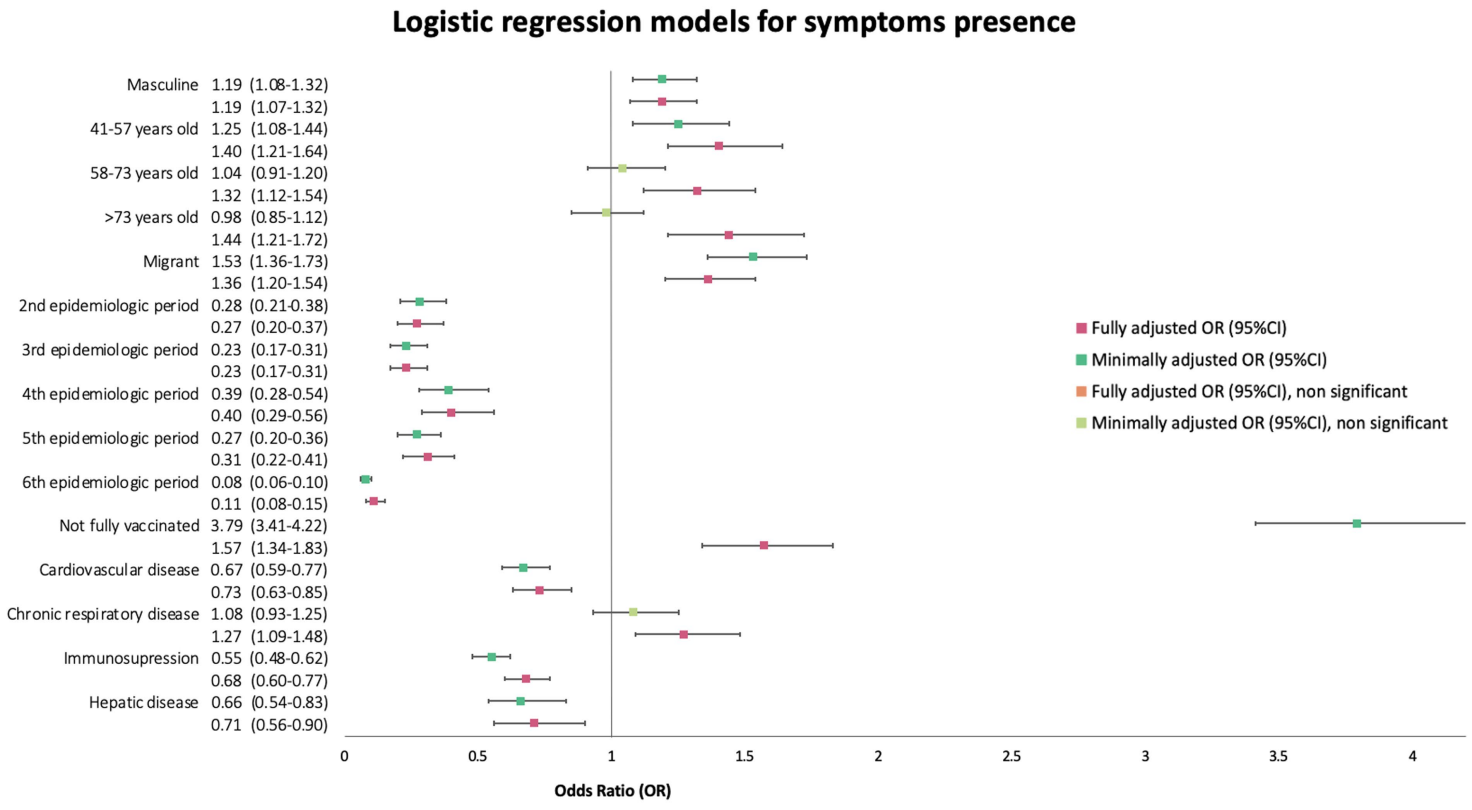
Forest plot of minimally (mOR) and fully (aOR) adjusted odds ratios with 95% confidence intervals (95%CI) for the presence of symptoms logistic regression.

Migrants had a minimally adjusted OR by sex and age quartile (mOR) of 1.53 (95%CI 1.36–1.73) and a fully adjusted OR (aOR) of 1.36 (95%CI 1.20–1.54). We adjusted the multivariate model for sex, age category, epidemiologic period of SARS-CoV-2 transmission, vaccination status, CVD, CRD, immunosuppressive condition, and hepatic disease (Figure 3).

**Figure 4:**
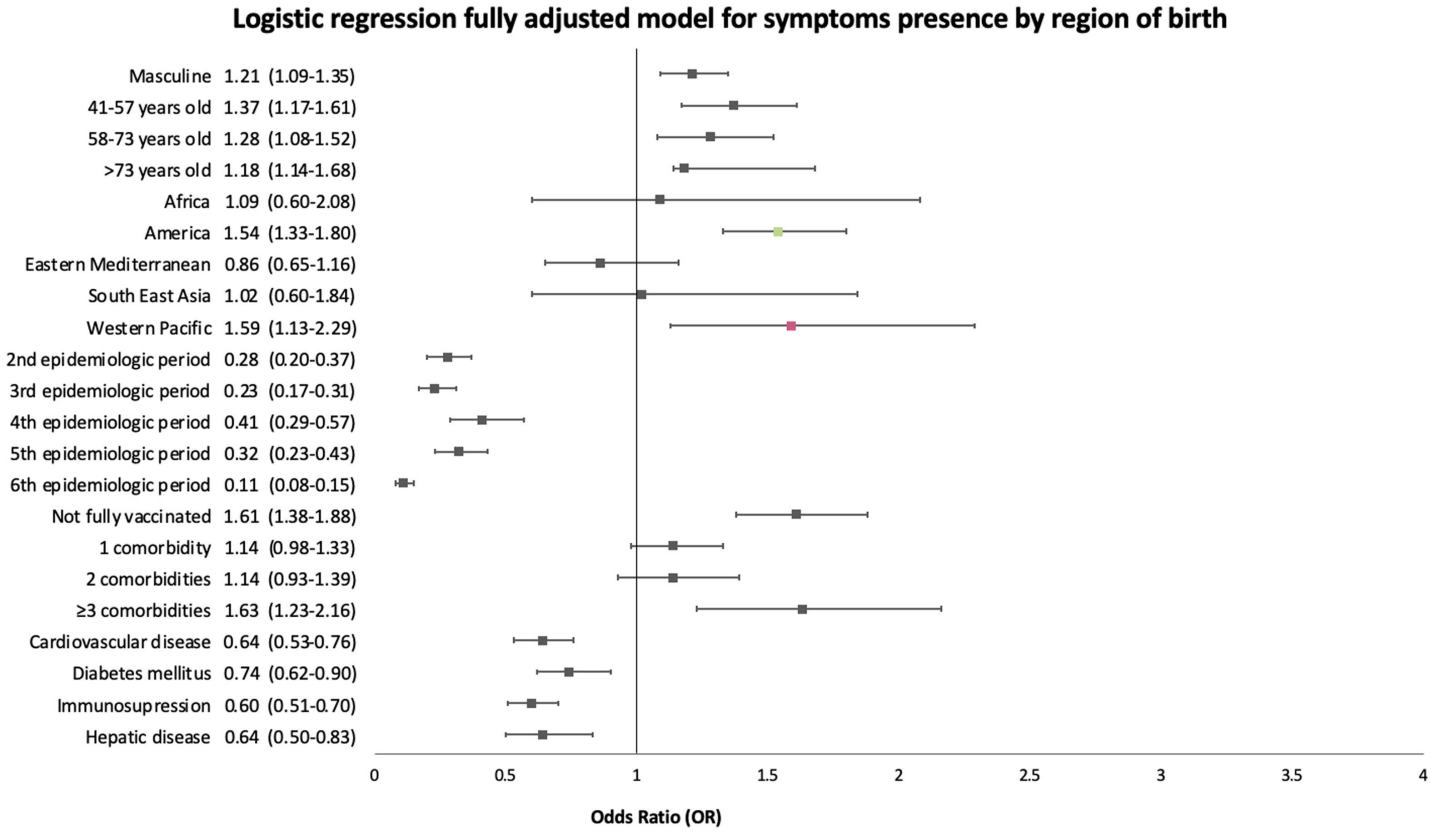
Forest plot of fully adjusted odds (aOR) ratios with 95% confidence intervals of the sub-analysis using region of birth as the explanatory variable for the presence of symptoms logistic regression.

We also performed a sub-analysis using region of birth as the explanatory variable instead of dichotomic migration status. The fully adjusted model for this outcome was adjusted by the covariates shown in Figure 4. It showed increased odds of presenting with symptoms at diagnosis only for patients born in the Americas Region (OR 1.54, 95%CI 1.33–1.80) and in the Western Pacific Region (OR 1.59, 95%CI 1.13–2.29) (Figure 4).

### 3.3 Hospitalisation

**Figure 5:**
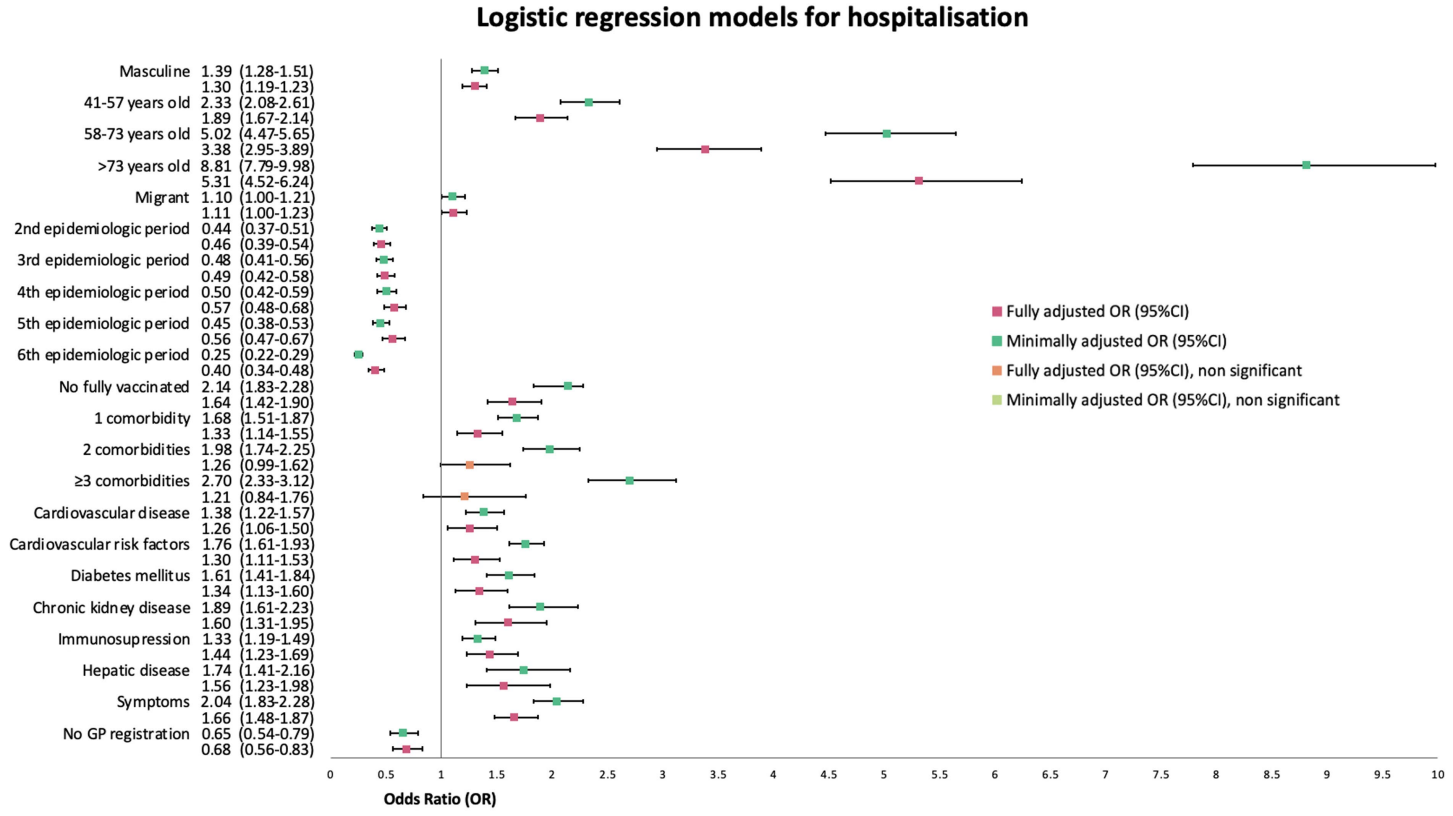
Forest plot of minimally (mOR) and fully (aOR) adjusted odds ratios with 95% confidence intervals (95%CI) for hospitalisation logistic regression.

When using a minimally adjusted model, migrants had a mOR of 1.10 (95%CI 1.00–1.21, *p* = 0.04). The change of the OR after fully adjusting the logistic regression model was minimal (aOR 1.11, 95%CI 1.00–1.23, p = 0.04). More information about the minimally adjusted model and the fully adjusted model for this outcome and the covariates used can be found in Figure 5.

**Figure 6:**
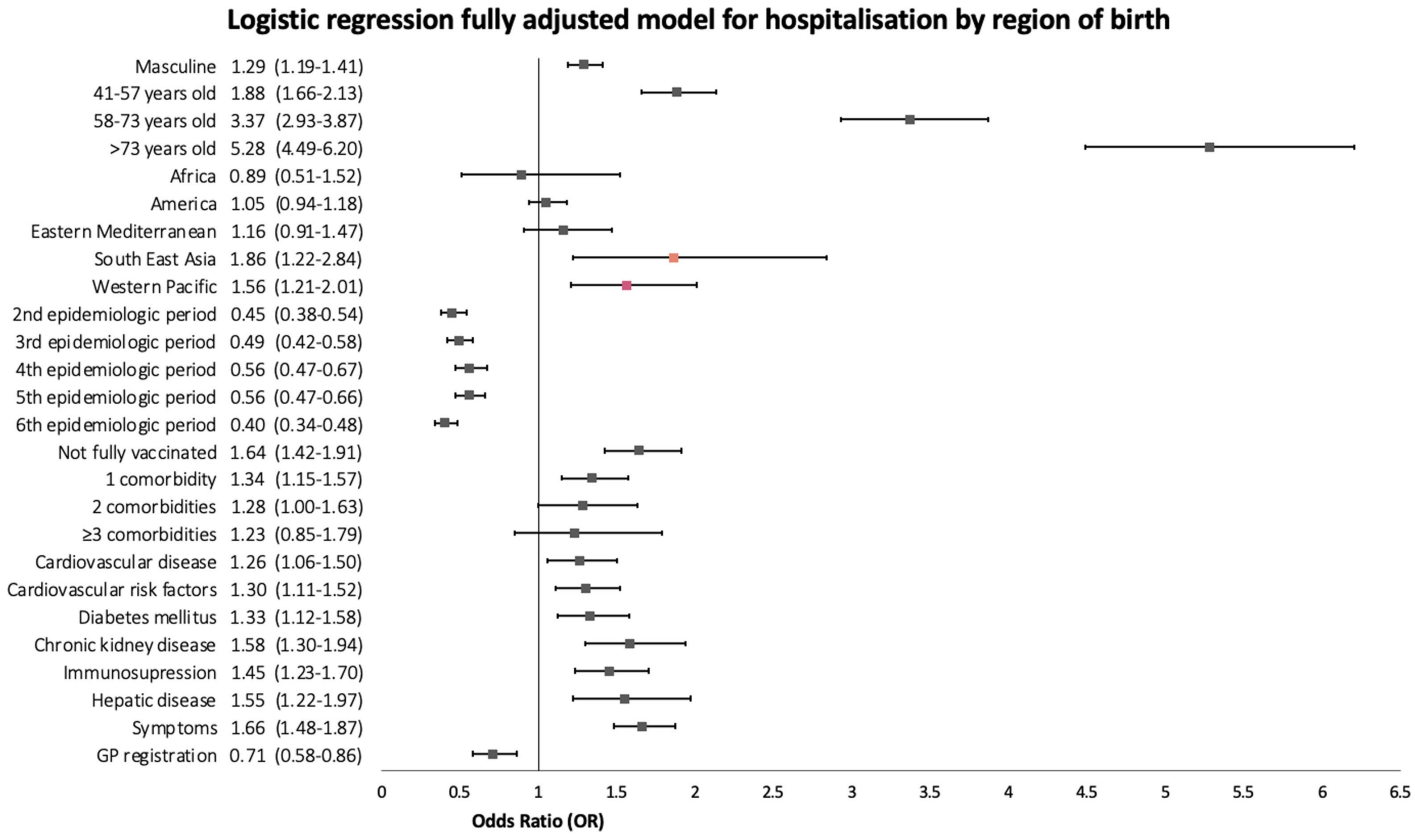
Forest plot of fully adjusted odds ratios with 95% confidence intervals of the sub-analysis using region of birth as the explanatory variable for hospitalisation logistic regression.

Subsequently, we performed a sub-analysis using the region of birth as the explanatory variable, which was adjusted by the same covariates. Only patients born in Southeast Asia and in the Western Pacific Region, had significantly increased odds of being hospitalised compared to patients born in Europe, with fully adjusted ORs (aOR) of 1.86 (95%CI 1.22–2.84, *p* = 0.004) and 1.56 (95%CI 1.21–2.01, *p* < 0.001), respectively (Figure 6).

### 3.4 Intensive care unit admission

**Figure 7:**
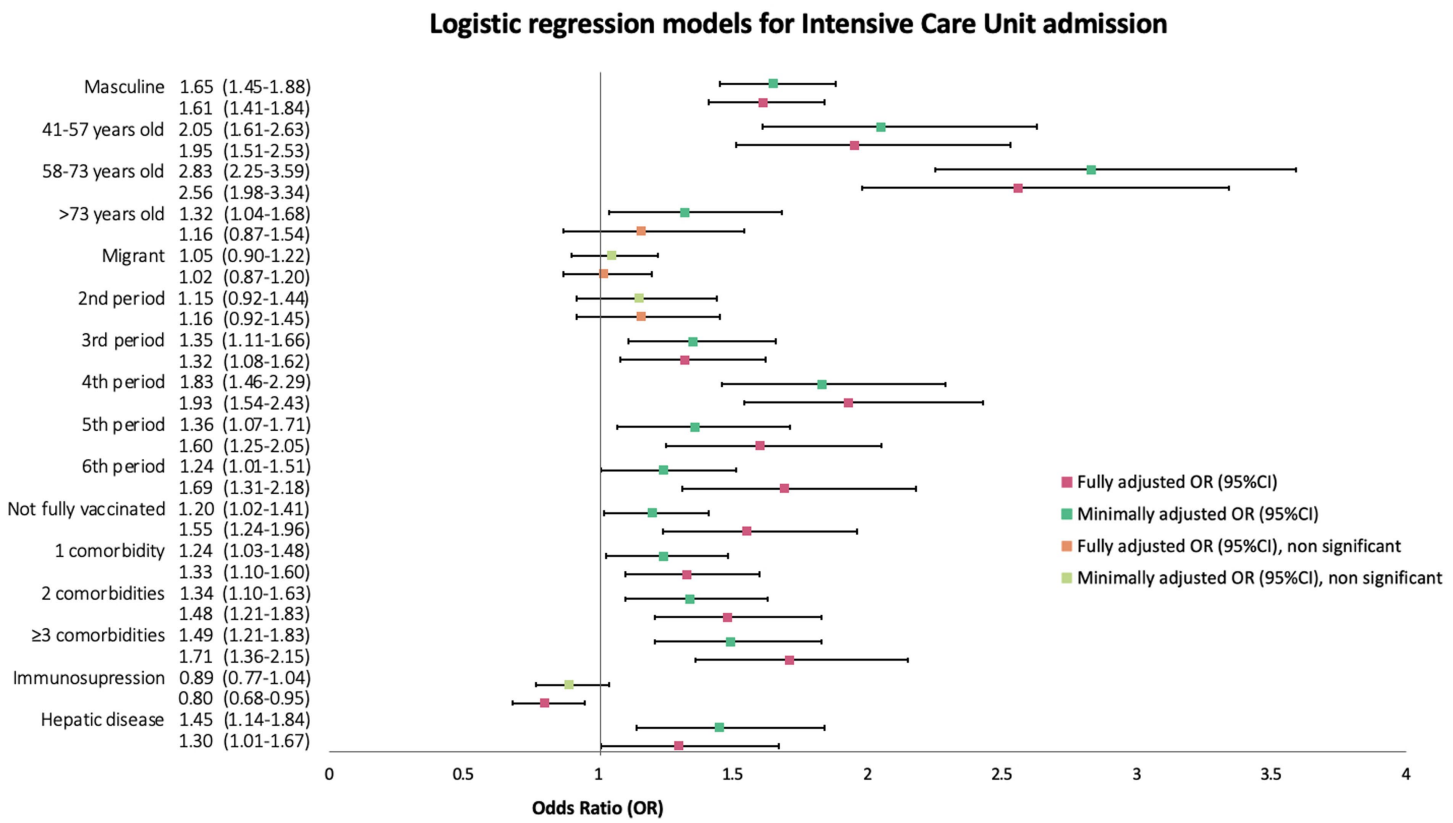
Forest plot of minimally (mOR) and fully (aOR) adjusted odds ratios with 95% confidence intervals (95%CI) for ICU admission logistic regression.

Minimally adjusted OR for migration status as an explanatory variable for ICU admission was 1.05 (95%IC, 0.90–1.22, *p* = 0.89). After fully adjusting a model, the aOR for migrants remained non-significant (aOR 1.02, 95%CI 0.87–1.20, *p* = 0.8). A detailed graphic account on odds ratio for all covariates considered can be found in Figure 7. Although a sub analysis by region of birth was performed and a multivariate regression model was also created, aOR of ICU admission was non-significant for all regions (data not shown).

### 3.5 Use of mechanical ventilation

**Figure 8:**
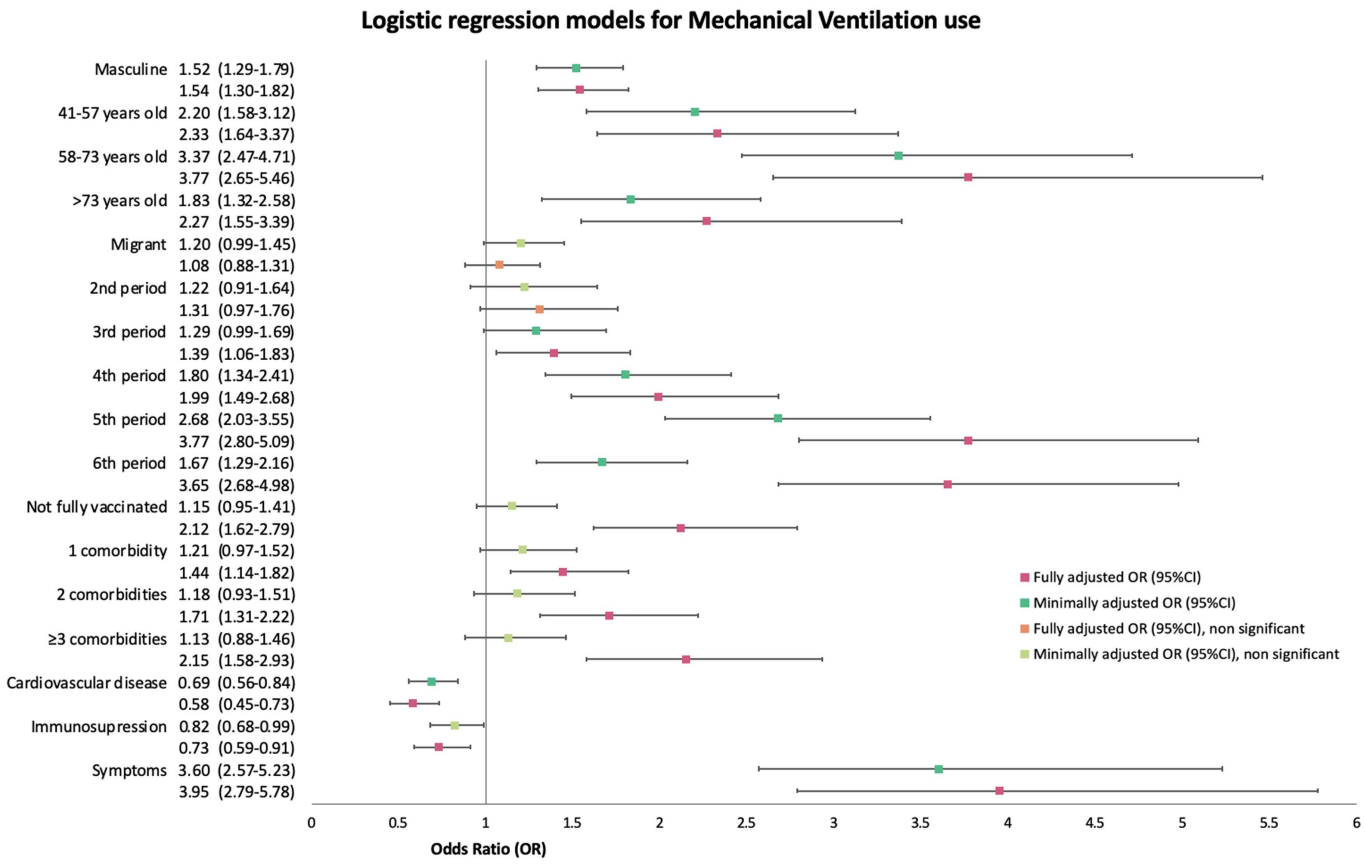
Forest plot of minimally (mOR) and fully (aOR) adjusted odds ratios with 95% confidence intervals (95%CI) for any type of mechanical ventilation use.

Similarly, to the outcome of ICU admission, neither the minimally adjusted nor the fully adjusted logistic regression for the use of any type of mechanical ventilation rendered increased or reduced odds for migrant patients compared to Spanish natives with an mOR 1.20 (95%CI 0.99–1.45, *p* = 0.06) of and an aOR of 1.08 (95%CI 0.88–1.31). A graphic representation of the bivariate and the multivariate model can be found in Figure 8. Sub-analysis by region of birth did not provide additional information, so data on this model is not shown.

### 3.6 In-hospital 30-day mortality

**Figure 9:**
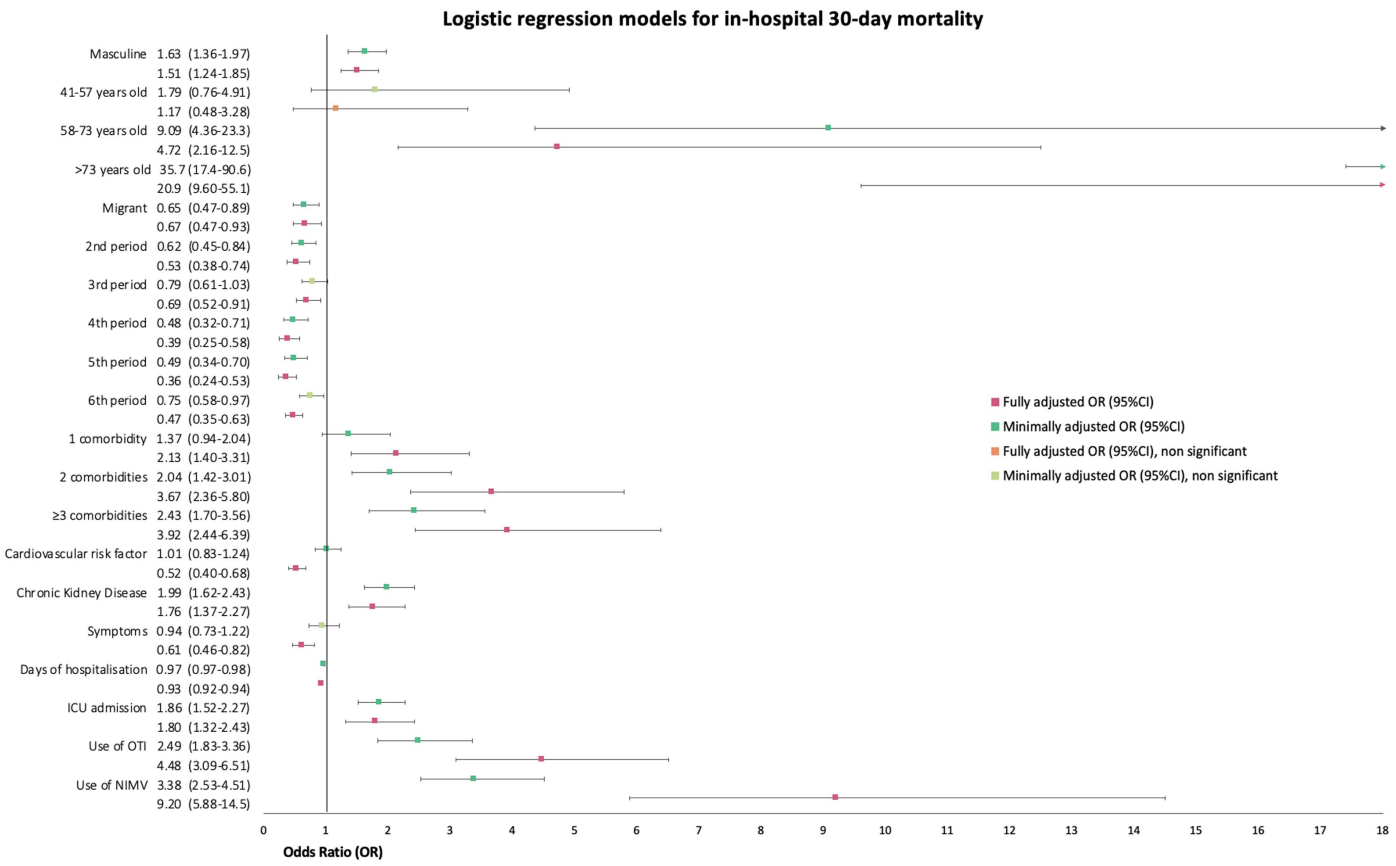
Forest plot of unadjusted and adjusted odds ratios (OR) with 95% confidence intervals (95%CI) for in-hospital 30-day mortality.

According to the minimally adjusted logistic regression, migrant patients were at reduced odds of in-hospital 30-day all-cause mortality with a mOR of 0.65 (95%CI 0.47–0.89, *p* = 0.01). When fully adjusted it rendered a similar OR of 0.67 (95%CI 0.47–0.93, *p* = 0.02) for migrant patients. A graphic summarising these models can be found in Figure 9. We also performed a sub-analysis considering the region of birth as the explanatory variable but found no significant results for patients of any region in comparison to European patients. Patients from the African region had an aOR of 0.84 (95CI% 0.13–3.31); patients from America an aOR of 0.71 (95CI% 0.45–1.08); patients from other European countries, an aOR of 0.67 (95CI% 0.22–1.61); patients from the South East Asia region, an aOR of 0.6 (95%CI 0.03–3.86); and patients from the Western Pacific had an aOR of 0.4 (95%CI 0.11–1.06).

## 4 Discussion

Our study found a higher risk of hospitalisation and symptomatology at onset for migrants. The obtained ORs for each of the outcomes did not change significantly between models minimally adjusted by sex and age quartiles and models fully adjusted by covariates. As mentioned earlier, research on migrant health disparities during the pandemic has shown highly conflicting results. While some found no significant differences in health outcomes between the native and migrant population, others stated that some groups of migrants, such as those coming from Latin America, had a higher risk of ICU admission, use of MV, or to a lower extent, IHM (14–24, 29). Our results were consistent with some other studies’ results on the subject, as we found that migrants had reduced odds of IHM and no significant differences in ICU admission or MV use. Although, the analysis did not show that migrants were at higher odds of these unfavourable health outcomes, it is important to acknowledge other important differences in our migrant population characteristics and in the odds of presenting with symptomatology and the need for hospitalisation.

Despite the widespread acknowledgement that migrants are at risk of worse health outcomes (16, 19–24), especially during times of crises, lower mortality and SARS-CoV-2 severity can be partially explained by the “healthy migrant effect,” a theory which has been thoroughly discussed in literature (4, 5, 13, 30–33). As in many other studies, we found that migrants were younger and had less comorbidities than natives, consistent with the abovementioned theory (13, 14, 19, 23, 24). It hypothesises that the majority of those who migrate, specially from low and middle-income countries to high-income countries, are the younger and healthier of the population. Most of these people are considered “economic migrants,” who usually migrate to work in developed countries, as a mean to improve their livelihoods (31). As many of the jobs they can get imply hard physical work, they are usually among the healthiest and youngest of their own societies (33). The low number of older migrants could partially explain some of the Odds Ratios’ wide confidence intervals in the multivariate analysis.

Our hospital is in a rather affluent neighbourhood of Barcelona city and, therefore, its referral population usually comes for nearby BHA, meaning most of them have a lower deprivation index. Our results show that migrant patients in Barcelona city tend to live in more deprived neighbourhoods (BHA). It is highly probable that if we studied migrant population from other hospitals’ referral areas, specially from smaller towns outside of Barcelona, the higher socioeconomic deprivation of migrants would be further evidenced, supporting the theory that an important proportion of them are “economic migrants” (31).

The results on the geographical distribution of migrant patients in this study are similar to those of other studies carried out in Spain that show that a higher proportion of migrants come from the Americas Region, specifically from Latin America (20–24, 30). This differs from the geographical origin of migrants living in other countries and could be explained by the shared language and historical colonial ties. The small number of patients born in certain regions also explain the OR’s wide confidence intervals in the sub-analyses by region.

We found that a third of the patients with a SARS-CoV-2 infection who visited our hospital were migrants, which is a higher percentage than the ones reported in other studies (21, 24, 30). It also represents a higher percentage than the one of the migrant populations living in our hospital’s referral area (34). A plausible explanation is that some patients living in other hospitals’ health areas with potentially higher migrant density could be referred to our centre because it is a high complexity hospital.

Important abovementioned differences in age and comorbidities could partially explain why fewer migrants were fully vaccinated at the time of the SARS-CoV-2 infection. Especially in the earlier vaccination campaigns, eligibility was based on advanced age and certain comorbidities, leading to the delay of young and healthy people’s vaccination to later stages of the pandemic. However, it is also important to consider barriers to healthcare access, lower acceptability of vaccines, and conflicting relationships with state institutions as potential limitations to getting vaccinated (13, 16, 35).

Although most patients, regardless of migration status, presented to the hospital with symptoms associated with a SARS-CoV-2 infection, it is important to notice that, after adjusting by sex, age, vaccination status, and relevant comorbidities, migrants were 1.36 times more likely to seek healthcare attention with symptoms compared with native patients, with an even higher OR in the population born in Latin America. There are very few studies that considered this as an outcome, possibly because it does not necessarily translate into more disease severity or need for hospitalisation; nonetheless, it is relevant. This difference in the presence of symptomatology could be due to a few reasons, one of them being that asymptomatic Spanish natives were more frequently diagnosed incidentally with SARS-CoV-2 infection because they were already linked to healthcare and being followed up for comorbidities or future interventions or procedures. This makes us wonder if migrants enjoy the same level of healthcare linkage and if their lower prevalence of chronic diseases could in fact be explained not because of good health but because of a lack of diagnosis and follow-up.

The fact that more natives are already registered with a GP and are familiar with how the health system works in Spain, could have also determined a higher use of primary healthcare services when developing mild symptomatology instead of going directly to a tertiary hospital. It could reflect a more selective use of high-complexity healthcare facilities to treat chronic or high-complexity pathologies rather than acute respiratory infections.

Several factors come in to play to explain the phenomenon of misuse of health services by migrant population, but their relationship with the health system at their countries of origin is a very important one. Many developing countries have inefficient primary healthcare facilities with many material and human resources shortcomings which is why patients tend to seek attention at larger health centres. Furthermore, for many people, only by migrating to a high-income country they can finally access free and quality healthcare, which could also explain the overuse of these services. Lack of trust and long-lasting relationship with primary health physicians should also be considered. There could also be difficulties in identifying what constitutes as an emergency. This is partially due to lower health literacy and lack of patients’ empowerment, concepts poorly explored and promoted in most developing countries (13, 16, 36).

We also reported slightly higher odds of being hospitalised for migrants. This is consistent with previously published research from other European countries (13, 15, 17, 18, 31, 36). These differences in hospitalisation for migrant patients could be attributed to a delay in seeking healthcare attention by migrants. This could be explained by a combination of factors, such as a lower level of health literacy, difficulties in recognising SARS-CoV-2 symptoms, problems to differentiate between health issues that require immediate attention and those which can be managed at home or by the GP. It is also important to mention that an important percentage of these patients could be considered “economic migrants” and that they tend to prioritise work commitments over their own health, as they fear losing their jobs or not being able to send money back home.

On the other hand, migrants could have fewer resources, worse home conditions, and insufficient social or family support to receive care at home, and therefore would need to be admitted even though the severity of the illness by itself does not require hospitalisation. The same is true for patients who would not be able to follow adequate preventive measures and social distance recommendations at home, either because of specific living conditions, overcrowding, or lack of knowledge and support. This could partially explain why there is a lower IHM among migrants when they require hospital admission more often. We should also consider other genetic and biological factors that are beyond the scope of this study to explain these kind of health outcomes discrepancies in migrant population.

### 4.1 Limitations

One of the most important issues was the accuracy of migration specific information. For example, we were unable to identify those migrants with refugee status, who are possibly at the highest risk of unfavourable health outcomes. The type of document used for registration at the hospital was used as a proxy for nationality, but we did not have information about the exact type of visa migrants had.

There was an important mismatch between nationality and country of birth. This could be due to the fact that a considerable percentage of migrants hold dual citizenship and also that Spanish laws allow migrants of certain nationalities (mainly former Spanish colonies) to request permanent residence and subsequent naturalisation after 2 years of living in Spain. We could not confirm how long ago a migrant with Spanish nationality had been naturalised. Although we decided to define migration status by the country of birth, instead of the nationality because we believe inequalities and vulnerability do not completely disappear after naturalisation, we do believe that legal status is a factor that should also be explored in migrants (30).

It is very difficult to gather patients’ data relevant to all intersectional factors that contribute towards health outcomes disparities. We recognise that there are several other characteristics that interact with migration status and that we could not get information on. Data on the country or region of birth does not adequately reflect the extent of structural inequalities or if a patient belongs to an underserved or marginalised community (30). It was not possible to assume other sociocultural factors related to migration status, such as language barriers, religion, education, or ethnicity. We believe this last factor to be a social determinant of health, based on genetic vulnerability and on discrimination (35). An important limitation to explore this subject is that, generally, in EU countries it is not possible to collect data on ethnic background. Although many studies use migrant status as a proxy for ethnicity, we believe they are different determinants (24–34, 36).

Although we collected data on the majority of patients’ BHA of residence, we were not able to consider the socioeconomic deprivation index for the multivariate analysis because there was information missing for a large percentage. It is possible that patients living in other cities of Catalonia, from whom we could not get exact information on BHA of residence, were more socioeconomically deprived. Characteristics of migrant patients described in this study largely differ from those of migrant communities living outside of Barcelona city, where there is a higher migrant density. Furthermore, as most patients in this study belong to our hospital’s referral area, which is located between neighbourhoods with less socioeconomic deprivation, the results in this study should not be extrapolated to all migrant communities living in Catalonia.

## 5 Conclusion

Our study’s results show that even though migrants have reduced odds of in-hospital mortality during SARS-CoV-2 infection, there are other important characteristics and outcomes that should be considered. The fact that migrants are more likely to present with symptomatology at onset and less likely to be diagnosed incidentally could translate into an inefficient use of health services, lower levels of healthcare linkage and follow-up, mistrust in health professionals, and lower health literacy. The higher odds of being hospitalised could also reflect the abovementioned issues, as well as a delay in seeking healthcare attention, lack of their own health’s prioritisation, difficulty in recognising symptoms of disease, lack of resources and support to receive care at home, and other genetic and biological factors that are beyond the scope of this study.

The results of this study further evidence the fact that even in states with inclusive, universal, and free healthcare systems, such as Spain, diverse inequalities in health outcomes persist for minorities. These disparities cannot be explained simply by socioeconomic status or limitations in access healthcare services because of migratory status or the elevated price of healthcare. Although we believe the findings of this study will be useful to evidence existing inequalities between native and migrant communities living in high-income countries, we also think that more research is needed to identify more clearly the underlying structural factors that determine these disparities. Furthermore, we strongly believe that there is enough evidence to direct more efforts into interventions and health policies that could help reduce these gaps in access to healthcare and health literacy in minorities and other vulnerable groups, such as migrants.

## Data availability statement

The data analysed in this study is subject to the following licenses/restrictions: This is a database created and maintained until this day for purposes of epidemiologic surveillance. Although the original database is anonymized it does contain information about infection dates, age, and specific comorbidities that would be better protected from the public. However, if it is necessary to make them publicly available we could ask for permission from the Ethics Committee. Requests to access these datasets should be directed to vperezm@clinic.cat.

## Ethics statement

The studies involving humans were approved by Research Ethics Committee of Hospital Clinic Barcelona. The studies were conducted in accordance with the local legislation and institutional requirements. The ethics committee/institutional review board waived the requirement of written informed consent for participation from the participants or the participants’ legal guardians/next of kin because Dissociated data was used so that no information included allows the identification of participants. Written informed consent for participation was waived for this study in accordance with the national legislation (Spain’s Organic Law 3/2018 of December 5 on the Protection of Personal Data and Guarantee of digital rights) and the institutional requirements.

## Author contributions

VP-M: Conceptualization, Data curation, Formal analysis, Investigation, Methodology, Software, Visualization, Writing – original draft, Writing – review & editing. MB: Conceptualization, Formal analysis, Methodology, Supervision, Validation, Writing – review & editing. LB-M: Conceptualization, Data curation, Formal analysis, Investigation, Methodology, Writing – review & editing. IT-R: Formal analysis, Methodology, Software, Supervision, Validation, Writing – review & editing. JG-G: Data curation, Investigation, Software, Writing – review & editing. AV: Conceptualization, Methodology, Supervision, Validation, Writing – review & editing.
